# ﻿*Sinosenecio yaanensis* (Asteraceae, Senecioneae), a new species from western Sichuan, China

**DOI:** 10.3897/phytokeys.262.161687

**Published:** 2025-09-04

**Authors:** Tianmeng Qu, Xinyi Zheng, Xinyu Chen, Yanru Zhang, Tonglin Zhang, Bo Li, Ke Huang, Zhixi Fu

**Affiliations:** 1 Key Laboratory of Land Resources Evaluation and Monitoring in Southwest, Sichuan Normal University, Ministry of Education, Chengdu 610066, China; 2 College of Life Sciences, Sichuan Normal University, Chengdu 610101, China; 3 Ya’an Ecological and Environment Monitoring Center, Ya’an, 625000, China; 4 Sichuan Ecological Environmental Monitoring Center, Chengdu 610091, China; 5 Sustainable Development Research Center of Resources and Environment of Western Sichuan, Sichuan Normal University, Chengdu, China

**Keywords:** Morphology, new species, Senecioneae, taxonomy

## Abstract

*Sinosenecio yaanensis* K.Huang & Z.X.Fu (Asteraceae) is described as a new species from Tianquan County, western Sichuan, China. It resembles *S. chienii* and *S. homogyniphyllus* in its scapigerous habit and palmately veined ovate to ovate-cordate radical leaves, but differs in several stable traits. Its leaves are subcoriaceous with whitish blotches adaxially and a purplish red abaxial surface, whereas *S. chienii* has submembranous blades that are pale green on the abaxial surface, while *S. homogyniphyllus* has smaller membranous leaves. *Sinosenecio yaanensis* produces 3–10 (vs. 2–9 in *S. chienii* and mostly solitary in *S. homogyniphyllus*) capitula, ca. 8 (vs. 10–12 and 8–17) ray florets, and lacks a pappus (vs. absent or rarely with short hairs). Phylogenetic analyses based on chloroplast genomes and ITS sequences place *S. yaanensis* within the *Sinosenecio*–tussilaginoid assemblage corresponding to the Tussilagininae subtribe, clustering with *S. chienii* and *S. homogyniphyllus*.

## ﻿Introduction

*Sinosenecio* B.Nord. is a genus in the tribe Senecioneae (Asteraceae), established several decades ago (Nordenstam 1978). The genus currently comprises 49 species, which are primarily distributed in central and southwestern China (Chen et al. 2011; Liu and Yang 2012; Liu et al. 2019; Zou et al. 2020; Chen et al. 2022; Peng et al. 2022a; Su et al. 2023a, b; Zhang et al. 2025). Species of this genus typically have subscapiform or leafy stems, palmately veined leaves (occasionally pinnately veined), capitula ranging from solitary to numerous, and generally campanulate involucres that mostly lack calyculate bracts (Jeffrey and Chen 1984; Peng et al. 2022a). Molecular phylogenetic studies have shown that *Sinosenecio* is a polyphyletic genus (Pelser et al. 2007; Wang et al. 2009; Liu 2010; Liu and Yang 2011a, b; Gong et al. 2016). The genus can be divided into two major lineages based on differences in basic chromosome number (x = 30 vs. x = 24 or rarely 13) and endothecial cell wall thickenings of anthers (strictly polarized vs. both polarized and radial), with the former closely related to the tussilaginoid genera and the latter to *Nemosenecio* and *Tephroseris* (Zhang et al. 2025).

In 2022, a botanical survey in Tianquan County, Ya’an City, western Sichuan, China, led to the discovery of an unknown species of *Sinosenecio*. The plants resemble *Sinosenecio chienii* and *S. homogyniphyllus* in being perennial herbs with rhizomes, palmately veined radical leaves that are ovate to ovate-cordate, and terminal corymbiform inflorescences. Morphological comparisons revealed consistent differences in stem indumentum, leaf texture, involucre shape, and ray floret morphology. These differences support its recognition as a new species, described here as *Sinosenecio yaanensis*. Its morphological and floral micromorphological traits are documented and compared with those of allied species. A phylogenetic analysis based on complete chloroplast genomes was conducted to clarify its placement within the genus.

## ﻿Material and methods

### ﻿Morphological analysis

The new species was collected on 29 April 2025 in Xingye Township, Tianquan County, Ya’an City, Sichuan Province, China. Both living material and herbarium specimens were examined. Morphological comparisons were conducted with closely related species, including *Sinosenecio chienii* and *S. homogyniphyllus*, based on taxonomic literature and herbarium images. Taxonomic descriptions follow the terminology of Chen et al. (2011) and Beentje (2016). The holotype of *S. yaanensis* is deposited in the herbarium of Sichuan Normal University (**SCNU**). The conservation status was preliminarily assessed following the IUCN Red List Categories and Criteria (IUCN 2024).

### ﻿Genome assembly and annotation

Total genomic DNA was extracted from silica-dried leaf tissue using the modified CTAB protocol (Doyle and Doyle 1987). The nrITS region of *S. yaanensis* was amplified and sequenced with primers ITS1 and ITS4 (Doyle and Doyle 1987) following the procedure of Peng et al. (2022a). Paired-end DNA libraries for the chloroplast genome were constructed following the Illumina DNA Library Preparation Guide (Allen et al. 2006). The complete chloroplast genome was sequenced on the Illumina HiSeq X platform (San Diego, CA, USA). High-quality reads were assembled using GetOrganelle v1.7.2 with default parameters (Jin et al. 2020). Genome annotations were manually inspected and adjusted in Geneious after annotation with CPGAVAS2 (Kearse et al. 2012; Shi et al. 2019). A circular map of the chloroplast genome was generated using Organellar Genome Draw (OGDRAW; https://chlorobox.mpimpgolm.mpg.de/OGDraw.html, accessed 6 June 2025) (Greiner et al. 2019). Cis-splicing and trans-splicing gene structures were identified and visualized using CPGView (http://47.96.249.172:16085/cpgview/view) (Liu et al. 2023). Various plastome characteristics, including gene length and GC content, were analyzed using CPJSdraw (Li et al. 2023). The chloroplast genome and ITS sequences of *S. yaanensis* were deposited in GenBank (http://www.ncbi.nlm.nih.gov/) under accession numbers PV748924 and PV946708, respectively.

### ﻿Phylogenetic analysis

Phylogenetic relationships of *S. yaanensis* were reconstructed using maximum likelihood (ML) based on complete chloroplast genomes and ITS sequences. The chloroplast dataset included 48 species selected following Liu et al. (2024), with *Anthriscus cerefolium* and *Kalopanax septemlobus* as outgroups (Suppl. material 1: table S1). The ITS dataset comprised 56 accessions, including 45 *Sinosenecio*, 4 *Nemosenecio*, 6 *Tephroseris*, and *Petasites tricholobus* as the outgroup (Suppl. material 1: table S2), following Zhang et al. (2025).

Sequences were aligned using MAFFT v7.520 with the auto strategy (Katoh and Standley 2013). ModelFinder (Kalyaanamoorthy et al. 2017) selected TVM+F+I+R4 and TIM3e+G4 as the best-fit models for the chloroplast and ITS datasets, respectively. Phylogenetic trees were inferred with IQ-TREE v2.3.6 (Minh et al. 2020), and branch support was assessed with 1,000 ultrafast bootstrap replicates, with UFBoot ≥95% regarded as strong support (Hoang et al. 2018). Trees were visualized and annotated in FigTree v1.4.4.

## ﻿Results

### ﻿Taxonomic treatment

#### ﻿ Sinosenecio yaanensis

Taxon classificationPlantaeAsteralesAsteraceae

K.Huang & Z.X.Fu sp. nov.

F214B32E-3B5A-52A3-B316-1A51183B3C52

urn:lsid:ipni.org:names:77368634-1

Figs 1, 2, 3

##### Type.

China • Sichuan Province, Ya’an City, Tianquan County, Xingye Township, 29°52'36.89"N, 102°45'14.70"E, elev. 1,500–1,600 m, growing in humid forest understory with shade, 29 April 2025 (fl.), *Ke Huang & Zhixi Fu 8550* (holotype: SCNU!) (Fig. 3).

**Figure 1.**
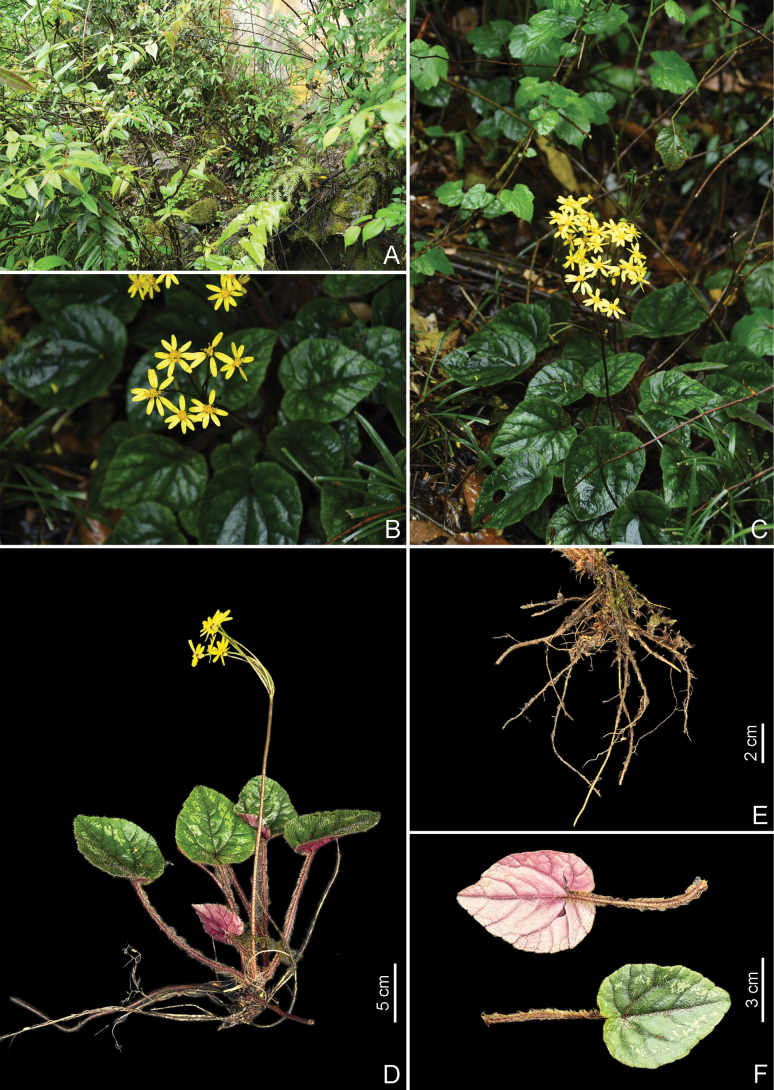
*Sinosenecio yaanensis*. A. Habitat; B–D. Habit; E. Rhizome; F. Adaxial (bottom) and abaxial (top) leaf surface (Photographed by Ke Huang and Z.X. Fu).

##### Diagnosis.

*Sinosenecio yaanensis* resembles *S. chienii* and *S. homogyniphyllus* in being scapigerous herbs with solitary, erect stems and palmately veined ovate to ovate-cordate radical leaves, but differs by its subcoriaceous blades with marginal whitish blotches adaxially and purplish red abaxially, densely fulvous-villous petioles, 3–10 capitula with about 8 ray florets bearing the longest ligules (11–12 mm), and achenes lacking a pappus (Table 1).

**Table 1.** Comparison between *Sinosenecio yaanensis*, *S. chienii* and *S. homogyniphyllus*.

Characters	*S. yaanensis*	*S. chienii*	*S. homogyniphyllus*
Height (cm)	18–35	20–30	10–30
Indumentum on stems	Sparsely pilose with white hairs	Fulvous-villous or ± glabrescent	Densely villous with long fulvous hairs
Indumentum on petiole	Densely fulvous villous	Fulvous-villous, ± glabrescent	Densely villous, with long fulvous hairs or sometimes subglabrous
Length of petiole (cm)	6–12	10–15	2.5–9
Shape of lamina	Ovate or ovate-cordate	Ovate or broadly ovate	Ovate to broadly ovate-orbicular, or reniform
Size of lamina (cm)	4.5–9 × 4–8.5	4–9 × 4.5–9.5	2–4 × 2.2–5.5
Texture of lamina	Subcoriaceous	Submembranous	Membranous
Color of lamina	Adaxially green with marginal whitish blotches, abaxially purplish red	Adaxially green to dark green, abaxially pale green or light purple when dry	Adaxially green, abaxially green or purple
Leaf margin	Entire with slight serration	Repand or sinuate-dentate, with prominent teeth	Repand-dentate or subentire, with obscure mucronulate teeth
Length of peduncle (cm)	3–8	2.5–7	2–3.5
Number of capitula	3–10	2–9	solitary or 2, rarely 7
Shape of involucre	Campanulate	Obconic-campanulate or narrowly campanulate	Obconic
Number of phyllaries	8–10	8–10	7–10, rarely 13
Number of ray florets	ca. 8	10–12	8–17
Ray size (mm)	11–12 × 3–4	8–10 × 2.5–3.5	9–9.5 × 3–3.5
Achene length (mm)	2.4–3	ca. 2.7	2–2.5
Pappus	Absent	Absent or rarely of several ca. 1.5 mm hairs	Absent, rarely of several hairs
Flowering	Apr–May	Apr–Jul	Apr-Jul
Fruiting	May–Jun	May–Aug	Jun-Aug
Distribution	Sichuan	Sichuan, Chongqing, Hunan	Sichuan, Hunan

##### Description.

***Scapigerous*** perennial herbs. ***Rhizomes*** short and stout, 1.5–3.5 mm in diam., with numerous fibrous roots. ***Stem*** solitary, erect, scapiform, 16–24 cm tall, simple, pale brown, sparsely pilose with white hairs. ***Leaves*** several, radical, rosulate, densely fulvous villous as the stems, long petiolate, petioles 6–12 cm long; blade ovate or ovate-cordate, with marginal whitish blotches, 4.5–9 × 4–8.5 cm, subcoriaceous; adaxially green, densely white-pubescent, especially along veins; abaxially purplish red, sparsely pubescent, with reddish-brown hairs on veins; margin villous; palmately veined, lateral veins 3–5 pairs, base cordate, margin entire with slight serration, apex acute. ***Capitula*** 3–10, 2–3 cm in diameter, arranged in a terminal corymb; peduncles slender, 3–8 cm long, green to pale brown, minutely and sparsely puberulent, with several linear bracts subtending the corymb. ***Receptacle*** slightly raised and pubescent. ***Involucres*** campanulate, 6–8 × 3–4 mm, ecalyculate; phyllaries 8–10, lanceolate, 6–7.5 × 1.5–2 mm; herbaceous, sparsely puberulent outside; green, apically purplish, acuminate. ***Ray florets*** ca. 8; corolla tube 1.3–2.3 mm long, glabrous; rays yellow, oblong, 11–12 × 3–4 mm, 4-veined, apically 3-denticulate. ***Disc florets*** numerous; corolla yellow, 4–5 mm long, with ca. 2–3 mm tube and campanulate limb; lobes ovate-oblong, ca. 1 mm long, apically acute. ***Anthers*** oblong, ca. 2 mm long, basally obtuse. ***Style*** branches ca. 0.5 mm long, recurved, apically truncate. ***Achenes*** cylindric, 2.4–3 mm long, smooth, glabrous. ***Pappus*** absent.

##### Floral micromorphological characters.

**Figure 2.**
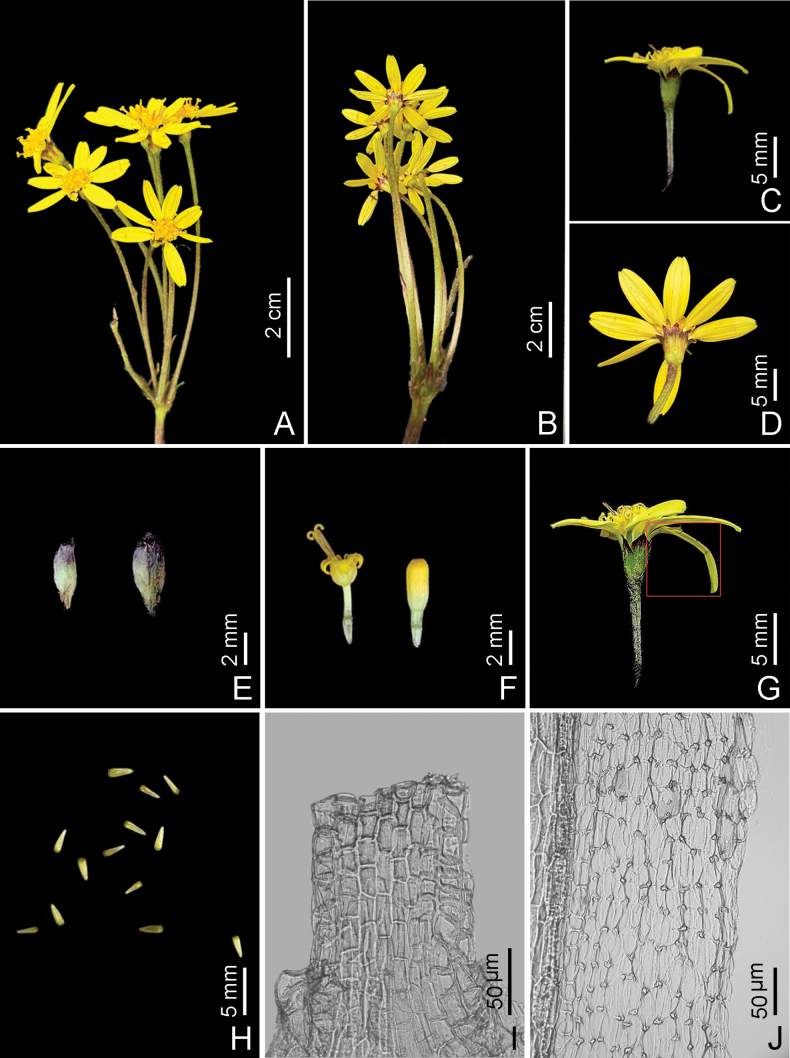
*Sinosenecio yaanensis*. A. Inflorescence (face view); B. Inflorescence (rear view); C. Capitulum(side view); D. Capitulum (rear view); E. Phyllaries; F. Disc florets; G. Ray floret (red box); H. Achenes; I. Uniformly-sized cells of filament collar; J. Strictly polarized anther endothecial cell wall thickenings (Photographed by T.M. Qu and Z.X. Fu).

The filament collar of *S. yaanensis* consisted of uniformly sized cells (Fig. 2I), and the anther endothecial cell wall thickenings were strictly polarized (Fig. 2J).

**Figure 3.**
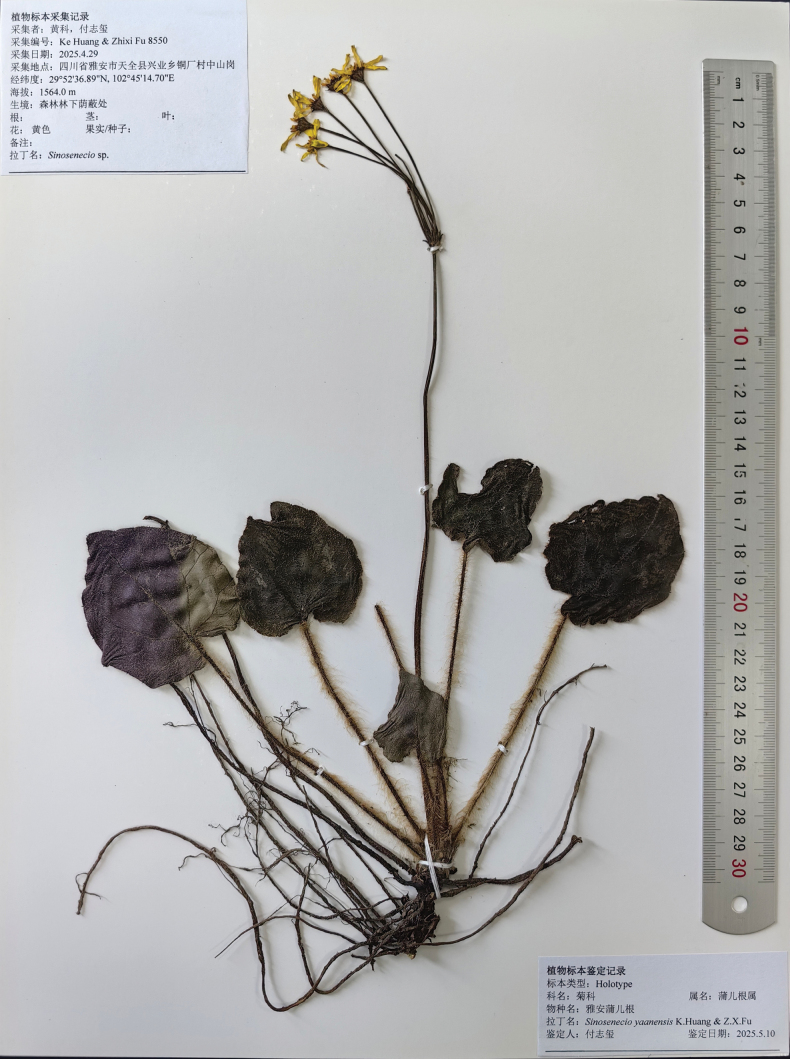
Holotype of *Sinosenecio yaanensis*.

##### Phenology.

Flowering from April to May; fruiting from May to June.

##### Etymology.

The specific epithet is derived from the type locality, Ya’an City, Sichuan Province, China. The proposed Chinese name is “雅安蒲儿根”, pronounced as “yǎ ān pú ér gēn”.

##### Distribution and ecology.

**Figure 4.**
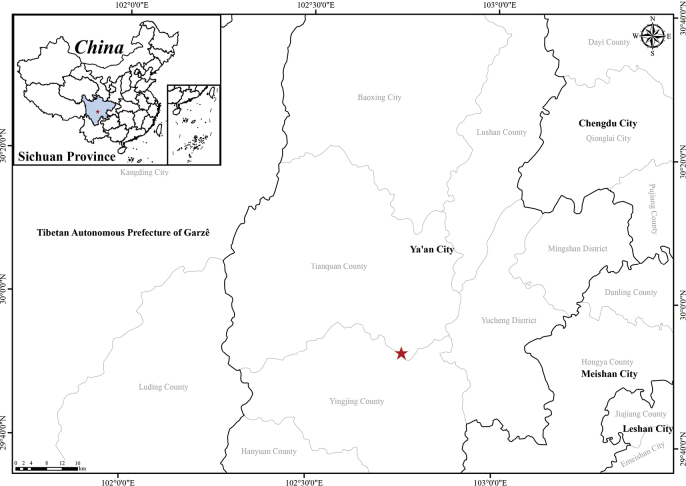
Distribution of *Sinosenecio yaanensis* sp. nov. (red star).

The new species is currently known only from its type locality, i.e. Tianquan county, Ya’an city in western Sichuan, China (Fig. 4). It grows in the humid, shaded forest understory at an altitude of 1,500–1,600 m above sea level.

##### Conservation status.

*Sinosenecio yaanensis* is currently recorded only from Tianquan County, Ya’an City, Sichuan Province, China. Field investigations revealed an estimated population of approximately 400–500 mature individuals confined to a narrow distribution range. According to the IUCN Red List Categories and Criteria (IUCN 2024), this meets the criteria for Vulnerable (VU) status under criterion D1, which applies to taxa with fewer than 1,000 mature individuals.

### ﻿Distributional differences

*Sinosenecio yaanensis* is currently known only from Tianquan County, Sichuan. In contrast, *S. chienii* and *S. homogyniphyllus* have broader and partly overlapping distributions. Both species occur in Tianquan, Baoxing, Mount Emei, Hongya, Dujiangyan, Leibo, and Zhangjiajie in Hunan. Additionally, *S. chienii* has been recorded in Shimian and Kangding (Sichuan), Wushan (Chongqing), and Shaoyang (Hunan), while *S. homogyniphyllus* is also found in Ebian and Meigu (Sichuan) (Suppl. material 1: table S3). These distribution records are based on specimens archived in the Chinese Virtual Herbarium (CVH, https://www.cvh.ac.cn/index.php, accessed 2 August 2025).

### ﻿Phylogenetic affiliation

**Figure 5.**
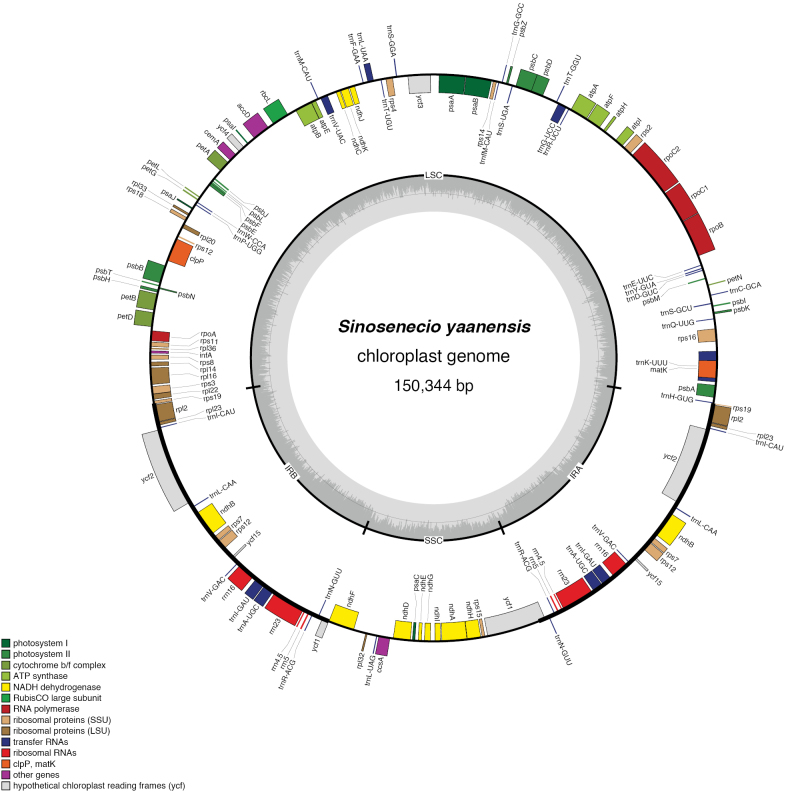
Circular map of *Sinosenecio yaanensis*. Genes inside of the circle are transcribed clockwise and those on the outside are transcribed counter-clockwise.

**Figure 6.**
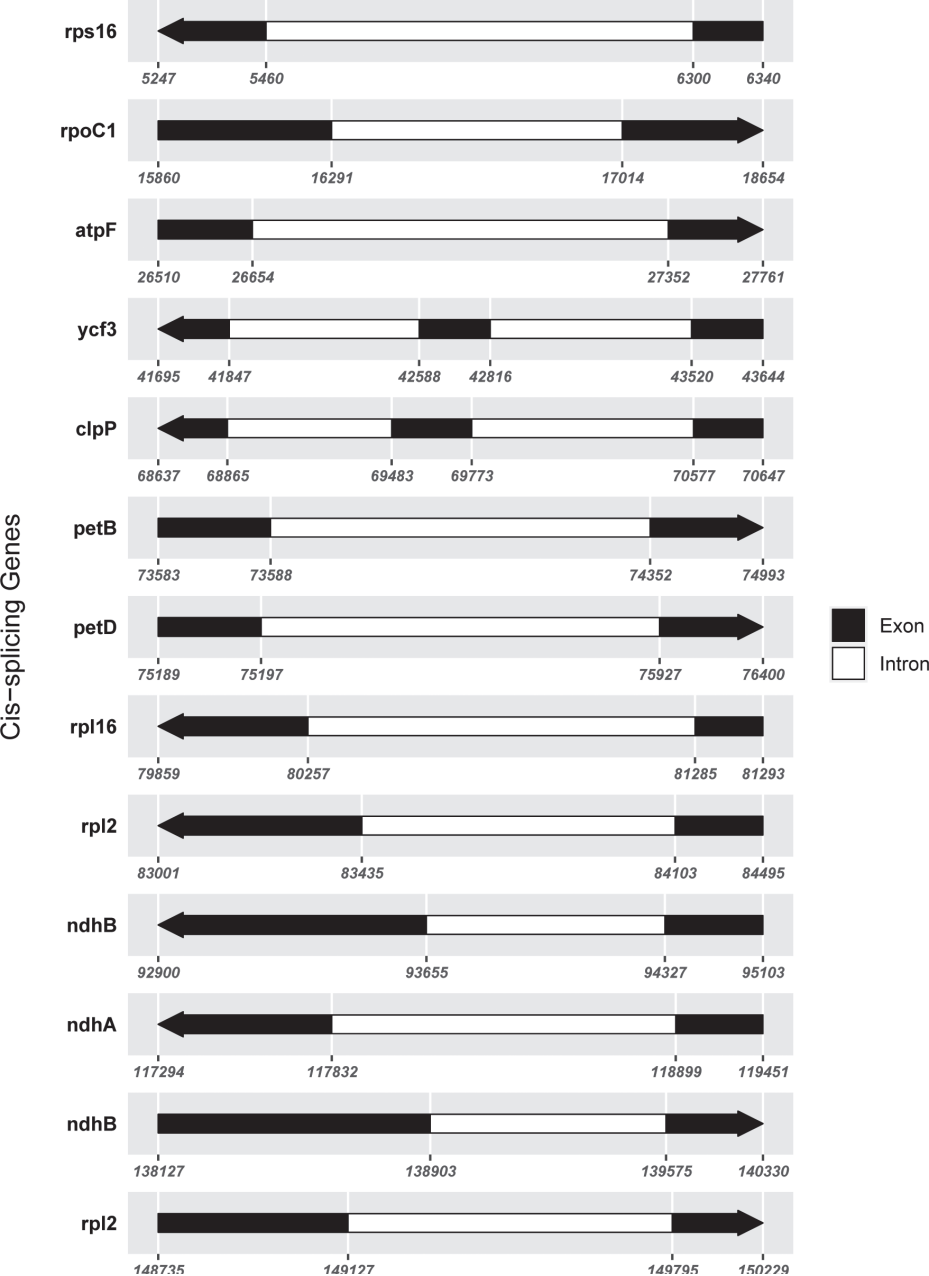
Schematic representation of the cis-splicing genes in the chloroplast genome of *Sinosenecio yaanensis*.

**Figure 7.**
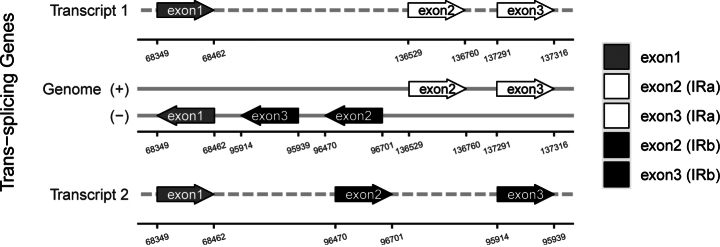
Schematic representation of the trans-splicing genes in the chloroplast genome of *Sinosenecio yaanensis*.

**Figure 8.**
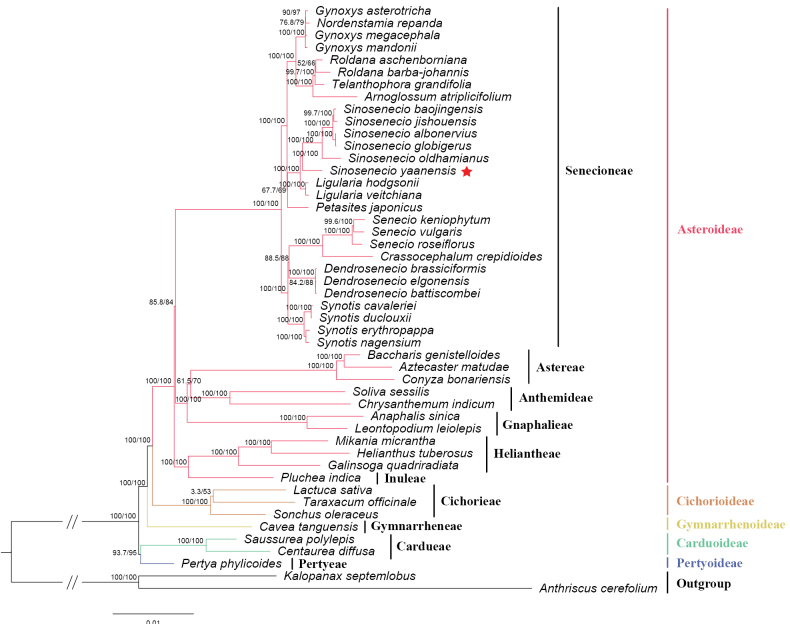
Phylogenetic tree reconstructed using the ML method based on complete chloroplast genomes of the Asteraceae. Node values represent SH-aLRT support (left) and bootstrap support (right) values. The phylogenetic position of *Sinosenecio yaanensis* is marked with a red star.

**Figure 9.**
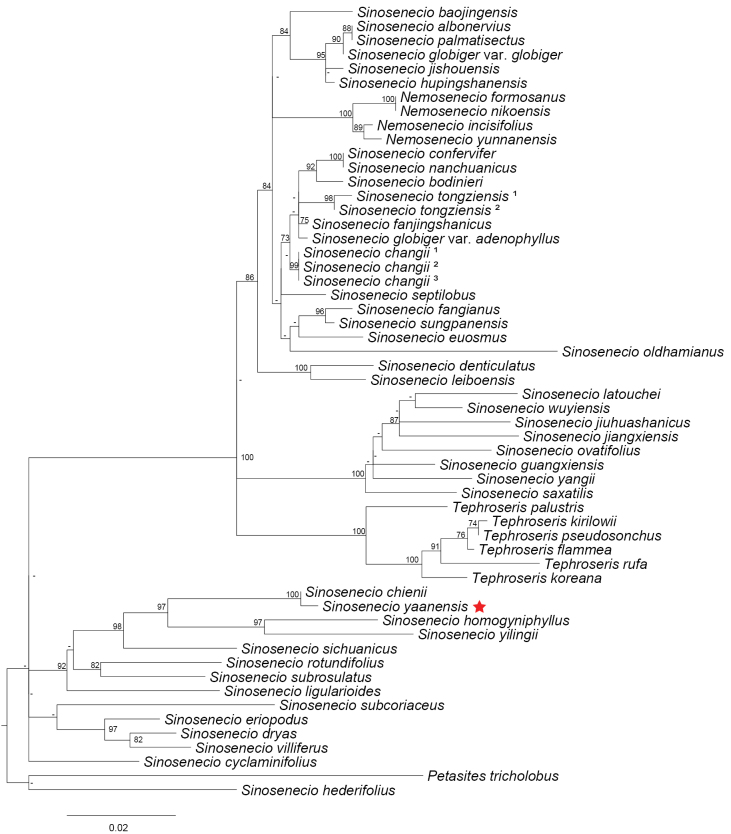
Phylogenetic tree reconstructed using the ML method based on ITS sequences. Node values represent bootstrap support values; values below 70 are indicated by “-”. The phylogenetic position of *Sinosenecio yaanensis* is marked with a red star.

The chloroplast genome of *S. yaanensis* is 150,344 bp in length with an overall GC content of 37.41%, and the IR regions have a GC content of 43.01% (Fig. 5, Table 2). The genome contains multiple cis-splicing genes and one trans-spliced gene, *rps12*, as illustrated in Figs 6, 7. The complete chloroplast genome was included in a phylogenetic analysis of *Sinosenecio* (Fig. 8). *S. yaanensis* is placed within a well-supported monophyletic lineage of *Sinosenecio*, separated from other genera of Senecioneae (Peng et al. 2022b; Liu et al. 2024). The ITS phylogeny (Fig. 9) places *S. yaanensis* within the *Sinosenecio*–tussilaginoid assemblage, a well-supported clade corresponding to the Tussilagininae subtribe (Liu 2010; Ren et al. 2017; Zhang et al. 2025). Within this assemblage, *S. yaanensis* forms a fully supported sister relationship with *S. chienii* (BS = 100). Together with *S. homogyniphyllus* and *S. yilingii*, they constitute a subclade with strong support (BS = 97) as a distinct group within the assemblage.

**Table 2.** Characteristics of the complete chloroplast genomes of *Sinosenecio yaanensis*.

Species	Genome Size (bp)	LSC Size (bp)	IRa/IRb Size (bp)	SSC Size (bp)	Total GC Content (%)	GC content in LSC (%)	GC content in IRa/IRb (%)	GC content in SSC (%)
*Sinosenecio yaanensis*	150, 344	82, 885	24, 639	18, 181	37.41	35.57	43.01	30.65

## Supplementary Material

XML Treatment for Sinosenecio yaanensis

## ﻿Additional information

### Use of AI

No use of AI was reported.

### Author ORCIDs

Tianmeng Qu https://orcid.org/0009-0008-0185-5306

Xinyi Zheng https://orcid.org/0009-0003-2300-5525

Xinyu Chen https://orcid.org/0009-0008-6347-2490

Yanru Zhang https://orcid.org/0009-0009-0450-2001

Tonglin Zhang https://orcid.org/0009-0000-8345-7097

Bo Li https://orcid.org/0000-0002-5131-8639

Ke Huang https://orcid.org/0009-0006-2057-5452

Zhixi Fu https://orcid.org/0000-0002-2789-6287

### Data availability

All of the data that support the findings of this study are available in the main text or Supplementary Information.

## Supplementary materials

Supplementary material 1 Supplementary information Data type docx Explanation note **table S1.** Species and GenBank accession numbers used in chloroplast phylogenetic analysis. **table S2.** Species and GenBank accession numbers used in ITS phylogenetic analysis. **table S3.** Voucher specimen records of *Sinosenecio chienii* and *S. homogyniphyllus* from the Chinese Virtual Herbarium (CVH). https://binary.pensoft.net/file/1409265This dataset is made available under the Open Database License (http://opendatacommons.org/licenses/odbl/1.0/). The Open Database License (ODbL) is a license agreement intended to allow users to freely share, modify, and use this Dataset while maintaining this same freedom for others, provided that the original source and author(s) are credited.Tianmeng Qu, Xinyi Zheng, Xinyu Chen, Yanru Zhang, Tonglin Zhang, Bo Li, Ke Huang, Zhixi Fu
