# Supplementary material for: ﻿Sinosenecio yaanensis (Asteraceae, Senecioneae), a new species from western Sichuan, China
Source: PhytoKeys. 2025 Sep 4;262:129–44. doi: 10.3897/phytokeys.262.161687 (PMC12426625; doi:10.3897/phytokeys.262.161687)
Supplement: Supplementary material 1 — Supplementary information [file phytokeys-262-129_article-161687__-s001.docx]

**Supplementary material**

**Supplementary Table 1.** Species and GenBank accession numbers used in chloroplast phylogenetic analysis.

|  | **Species** | **GenBank numbers** | **Subfamily** | **Tribe** |
| --- | --- | --- | --- | --- |
| 1 | *Anthriscus cerefolium* | GU456628 | Apioideae | Scandiceae |
| 2 | *Kalopanax septemlobus* | NC022814 | Aralioideae | Aralieae |
| 3 | *Chrysanthemum indicum* | MH165290 | Asteroideae | Anthemideae |
| 4 | *Soliva sessilis* | NC034851 | Asteroideae | Anthemideae |
| 5 | *Aztecaster matudae* | NC034898 | Asteroideae | Astereae |
| 6 | *Baccharis genistelloides* | NC034852 | Asteroideae | Astereae |
| 7 | *Conyza bonariensis* | NC035884 | Asteroideae | Astereae |
| 8 | *Anaphalis sinica* | NC034648 | Asteroideae | Gnaphalieae |
| 9 | *Leontopodium leiolepis* | NC027835 | Asteroideae | Gnaphalieae |
| 10 | *Galinsoga quadriradiata* | NC031853 | Asteroideae | Heliantheae |
| 11 | *Helianthus tuberosus* | MG696658 | Asteroideae | Heliantheae |
| 12 | *Mikania micrantha* | NC031833 | Asteroideae | Heliantheae |
| 13 | *Pluchea indica* | NC038194 | Asteroideae | Inuleae |
| 14 | *Arnoglossum atriplicifolium* | MK170176 | Asteroideae | Senecioneae |
| 15 | *Crassocephalum crepidioides* | MW362305 | Asteroideae | Senecioneae |
| 16 | *Dendrosenecio battiscombei* | KY434195 | Asteroideae | Senecioneae |
| 17 | *Dendrosenecio brassiciformis* | MG560051 | Asteroideae | Senecioneae |
| 18 | *Dendrosenecio elgonensis* | KY434194 | Asteroideae | Senecioneae |
| 19 | *Gynoxys asterotricha* | MK044798 | Asteroideae | Senecioneae |
| 20 | *Gynoxys mandonii* | MK056106 | Asteroideae | Senecioneae |
| 21 | *Gynoxys megacephala* | MN328892 | Asteroideae | Senecioneae |
| 22 | *Ligularia hodgsonii* | NC039381 | Asteroideae | Senecioneae |
| 23 | *Ligularia veitchiana* | NC039385 | Asteroideae | Senecioneae |
| 24 | *Nordenstamia repanda* | MK086040 | Asteroideae | Senecioneae |
| 25 | *Petasites japonicus* | MN385243 | Asteroideae | Senecioneae |
| 26 | *Roldana aschenborniana* | MK170177 | Asteroideae | Senecioneae |
| 27 | *Roldana barba-johannis* | MK170178 | Asteroideae | Senecioneae |
| 28 | *Senecio keniophytum* | MH483946 | Asteroideae | Senecioneae |
| 29 | *Senecio roseiflorus* | MH483948 | Asteroideae | Senecioneae |
| 30 | *Senecio vulgaris* | NC046693 | Asteroideae | Senecioneae |
| 31 | *Sinosenecio albonervius* | OL678114 | Asteroideae | Senecioneae |
| 32 | *Sinosenecio baojingensis* | MZ325394 | Asteroideae | Senecioneae |
| 33 | *Sinosenecio globigerus* | OR752442 | Asteroideae | Senecioneae |
| 34 | *Sinosenecio jishouensis* | MT876597 | Asteroideae | Senecioneae |
| 35 | *Sinosenecio oldhamianus* | MN013404 | Asteroideae | Senecioneae |
| 36 | *Sinosenecio yaanensis* | PV748924 | Asteroideae | Senecioneae |
| 37 | *Synotis cavaleriei* | OM912601 | Asteroideae | Senecioneae |
| 38 | *Synotis duclouxii* | OM912602 | Asteroideae | Senecioneae |
| 39 | *Synotis erythropappa* | OQ985056 | Asteroideae | Senecioneae |
| 40 | *Synotis nagensium* | OM912603 | Asteroideae | Senecioneae |
| 41 | *Telanthophora grandifolia* | MK170179 | Asteroideae | Senecioneae |
| 42 | *Centaurea diffusa* | NC024286 | Carduoideae | Cardueae |
| 43 | *Saussurea polylepis* | NC036490 | Carduoideae | Cardueae |
| 44 | *Lactuca sativa* | AP007232 | Cichorioideae | Cichorieae |
| 45 | *Sonchus oleraceus* | NC048452 | Cichorioideae | Cichorieae |
| 46 | *Taraxacum officinale* | KX198561 | Cichorioideae | Cichorieae |
| 47 | *Cavea tanguensis* | MN473457 | Gymnarrhenoideae | Gymnarrheneae |
| 48 | *Pertya phylicoides* | NC057464 | Pertyoideae | Pertyeae |

**Supplementary Table 2.** Species and GenBank accession numbers used in ITS phylogenetic analysis.

|  | **Species** | **GenBank numbers** |
| --- | --- | --- |
| 1 | *Nemosenecio formosanus* | KU696044 |
| 2 | *Nemosenecio incisifolius* | KU696045 |
| 3 | *Nemosenecio nikoensis* | EF538265 |
| 4 | *Nemosenecio yunnanensis* | KU696047 |
| 5 | *Petasites tricholobus* | MH808185 |
| 6 | *Sinosenecio albonervius* | KU696079 |
| 7 | *Sinosenecio baojingensis* | KU696080 |
| 8 | *Sinosenecio bodinieri* | KU696081 |
| 9 | *Sinosenecio changii* | KT149885 |
| 10 | *Sinosenecio changii* | KT150040 |
| 11 | *Sinosenecio changii* | KU696082 |
| 12 | *Sinosenecio chienii* | KU696084 |
| 13 | *Sinosenecio confervifer* | KT149891 |
| 14 | *Sinosenecio cyclaminifolius* | KU696087 |
| 15 | *Sinosenecio denticulatus* | KU696088 |
| 16 | *Sinosenecio dryas* | KU696089 |
| 17 | *Sinosenecio eriopodus* | KT150043 |
| 18 | *Sinosenecio euosmus* | GU818710 |
| 19 | *Sinosenecio fangianus* | KU696094 |
| 20 | *Sinosenecio fanjingshanicus* | KT149887 |
| 21 | *Sinosenecio globiger* var. *globiger* | KT149897 |
| 22 | *Sinosenecio globiger* var. *adenophyllus* | KU696097 |
| 23 | *Sinosenecio guangxiensis* | KU696110 |
| 24 | *Sinosenecio hederifolius* | KU696111 |
| 25 | *Sinosenecio homogyniphyllus* | KU696114 |
| 26 | *Sinosenecio hupingshanensis* | KU696115 |
| 27 | *Sinosenecio jiangxiensis* | KT149881 |
| 28 | *Sinosenecio jishouensis* | KU696116 |
| 29 | *Sinosenecio jiuhuashanicus* | KU696117 |
| 30 | *Sinosenecio latouchei* | JF978612 |
| 31 | *Sinosenecio leiboensis* | KU696118 |
| 32 | *Sinosenecio ligularioides* | KU696119 |
| 33 | *Sinosenecio nanchuanicus* | KT149895 |
| 34 | *Sinosenecio oldhamianus* | JF978618 |
| 35 | *Sinosenecio ovatifolius* | MT522620 |
| 36 | *Sinosenecio palmatisectus* | KU696125 |
| 37 | *Sinosenecio rotundifolius* | KU696126 |
| 38 | *Sinosenecio saxatilis* | KT149882 |
| 39 | *Sinosenecio septilobus* | JF978624 |
| 40 | *Sinosenecio sichuanicus* | KU696127 |
| 41 | *Sinosenecio subcoriaceus* | KU696128 |
| 42 | *Sinosenecio subrosulatus* | KU696129 |
| 43 | *Sinosenecio sungpanensis* | KU696130 |
| 44 | *Sinosenecio tongziensis* | PP556465 |
| 45 | *Sinosenecio tongziensis* | PP556466 |
| 46 | *Sinosenecio villiferus* | KU696131 |
| 47 | *Sinosenecio wuyiensis* | KT149878 |
| 48 | *Sinosenecio yaanensis* | PV946708 |
| 49 | *Sinosenecio yangii* | OM413747 |
| 50 | *Sinosenecio yilingii* | KU696132 |
| 51 | *Tephroseris flammea* | KU696137 |
| 52 | *Tephroseris kirilowii* | MH711065 |
| 53 | *Tephroseris koreana* | KU696138 |
| 54 | *Tephroseris palustris* | MW779519 |
| 55 | *Tephroseris pseudosonchus* | KU696139 |
| 56 | *Tephroseris rufa* | AY176166 |

**Supplementary Table 3 .** Voucher specimen records of *Sinosenecio chienii* and *S. homogyniphyllus* from the Chinese Virtual Herbarium (CVH).

| **Species** | **Locality** | **Collection Date** | **Voucher No.** | **Herbarium** |
| --- | --- | --- | --- | --- |
| Sinosenecio chienii | Tianquan, Sichuan | 20090515 | 01894856 | PE |
|  | Tianquan, Sichuan | 20090515 | 01894855 | PE |
|  | Hongya, Sichuan | 20090521 | 01894801 | PE |
|  | Mount Emei, Sichuan | 19710716 | 01836290 | PE |
|  | Mount Emei, Sichuan | 19950522 | 01836258 | PE |
|  | Mount Emei, Sichuan | 19950517 | 01830169 | PE |
|  | Tianquan, Sichuan | 19590610 | 00853028 | PE |
|  | Tianquan, Sichuan | 19550424 | 00853026 | PE |
|  | Tianquan, Sichuan | 19590605 | 00853023 | PE |
|  | Mount Emei, Sichuan | 19410412 | 00853022 | PE |
|  | Mount Emei, Sichuan | 19420420 | 00853020 | PE |
|  | Mount Emei, Sichuan | 19640514 | 00853021 | PE |
|  | Mount Emei, Sichuan | 19640514 | 00853018 | PE |
|  | Tianquan, Sichuan | 19530331 | 00852814 | PE |
|  | Wushan, Chongqing | 19580427 | 00852811 | PE |
|  | Mount Emei, Sichuan | 19550622 | 00852806 | PE |
|  | Shaoyang, Hunan | 19960928 | 00852805 | PE |
|  | Mount Emei, Sichuan | 19950517 | 01830169 | PE |
|  | Mount Emei, Sichuan | 19420420 | 0618359 | IBSC |
|  | Tianquan, Sichuan | 19530331 | 0618355 | IBSC |
|  | Mount Emei, Sichuan | 19410412 | 0618362 | IBSC |
|  | Mount Emei, Sichuan | 19410419 | 0618363 | IBSC |
|  | Zhangjiajie, Hunan | 19910620 | 0618365 | IBSC |
|  | Mount Emei, Sichuan | 19570507 | 0618357 | IBSC |
|  | Tianquan, Sichuan | 19360502 | 0618360 | IBSC |
|  | Tianquan, Sichuan | 19530424 | 0618356 | IBSC |
|  | Tianquan, Sichuan | 19800419 | 0618358 | IBSC |
|  | Tianquan, Sichuan | 19360412 | 0618364 | IBSC |
|  | Tianquan, Sichuan | 19530418 | 0618354 | IBSC |
|  | Tianquan, Sichuan | 19800428 | 0618353 | IBSC |
|  | Mount Emei, Sichuan | 19520509 | 0618352 | IBSC |
|  | Wushan, Chongqing | 19580427 | 00282916 | IBK |
|  | Tianquan, Sichuan | 19800419 | 0146706 | CDBI |
|  | Tianquan, Sichuan | 19800428 | 0146707 | CDBI |
|  | Tianquan, Sichuan | 19800428 | 0146708 | CDBI |
|  | Tianquan, Sichuan | 19800419 | 0146709 | CDBI |
|  | Tianquan, Sichuan | 19590605 | 0146710 | CDBI |
|  | Tianquan, Sichuan | 19610507 | 0146712 | CDBI |
|  | Baoxing, Sichuan | 19830420 | 0149507 | CDBI |
|  | Leibo, Sichuan | 19830524 | 0149596 | CDBI |
|  | Hongya, Sichuan | 19940627 | 0150593 | CDBI |
|  | Hongya, Sichuan | 19940627 | 0150594 | CDBI |
|  | Hongya, Sichuan | 19930608 | 0150665 | CDBI |
|  | Hongya, Sichuan | 19940608 | 0150671 | CDBI |
|  | Hongya, Sichuan | 19940608 | 0150672 | CDBI |
|  | Hongya, Sichuan | 19930602 | 0151155 | CDBI |
|  | Hongya, Sichuan | 19930602 | 0151156 | CDBI |
|  | Leibo, Sichuan | 19830529 | 0150987 | CDBI |
|  | Leibo, Sichuan | 19830524 | 0151001 | CDBI |
|  | Leibo, Sichuan | 19830529 | 0151003 | CDBI |
|  | Leibo, Sichuan | 19830524 | 0151004 | CDBI |
|  | Kangding, Sichuan | 20170814 | 0252165 | CDBI |
|  | Kangding, Sichuan | 20170814 | 0252164 | CDBI |
|  | Shimian, Sichuan | 20200615 | 0274536 | CDBI |
|  | Dujiangyan, Sichuan | 19870730 | 004825 | WCSBG |
|  | Dujiangyan, Sichuan | 20000523 | 004826 | WCSBG |
|  | Dujiangyan, Sichuan | 19870730 | 004827 | WCSBG |
|  | Mount Emei, Sichuan | 20100523 | 0172291 | HIB |
| *Sinosenecio homogyniphyllus* | Ebian, Sichuan | 20080918 | 02113488 | PE |
|  | Hongya, Sichuan | 20070624 | 01956055 | PE |
|  | Baoxing, Sichuan | 20070626 | 01956047 | PE |
|  | Baoxing, Sichuan | 20090521 | 01894854 | PE |
|  | Tianquan, Sichuan | 20090515 | 01894853 | PE |
|  | Hongya, Sichuan | 20070623 | 01894848 | PE |
|  | Hongya, Sichuan | 20070623 | 01894847 | PE |
|  | Hongya, Sichuan | 20070624 | 01894846 | PE |
|  | Hongya, Sichuan | 20070624 | 01894845 | PE |
|  | Baoxing, Sichuan | 20070626 | 01894843 | PE |
|  | Hongya, Sichuan | 20070624 | 01894844 | PE |
|  | Baoxing, Sichuan | 20070626 | 01894842 | PE |
|  | Baoxing, Sichuan | 20070626 | 01894841 | PE |
|  | Leibo, Sichuan | 19830420 | 01836288 | PE |
|  | Leibo, Sichuan | 19840419 | 01836289 | PE |
|  | Leibo, Sichuan | 19830420 | 01836287 | PE |
|  | Leibo, Sichuan | 19830529 | 01836286 | PE |
|  | Leibo, Sichuan | 19830529 | 01836285 | PE |
|  | Dujiangyan, Sichuan | 19870512 | 01717464 | PE |
|  | Tianquan, Sichuan | 19590615 | 00853000 | PE |
|  | Leibo, Sichuan | 19590609 | 00852996 | PE |
|  | Tianquan, Sichuan | 19590614 | 00852994 | PE |
|  | Tianquan, Sichuan | 19590615 | 00852995 | PE |
|  | Meigu, Sichuan | 19600614 | 00852993 | PE |
|  | Mount Emei, Sichuan | 19570612 | 00852992 | PE |
|  | Mount Emei, Sichuan | 19570607 | 00852991 | PE |
|  | Zhangjiajie, Hunan | 19750623 | 00852984 | PE |
|  | Tianquan, Sichuan | 19510621 | 00852988 | PE |
|  | Mount Emei, Sichuan | 19350406 | 00852986 | PE |
|  | Mount Emei, Sichuan | 19570607 | 0618411 | IBSC |
|  | Mount Emei, Sichuan | 19400628 | 0618413 | IBSC |
|  | Mount Emei, Sichuan | 19570612 | 0618412 | IBSC |
|  | Leibo, Sichuan | 19640424 | 0146765 | CDBI |
|  | Leibo, Sichuan | 19640530 | 0146764 | CDBI |
|  | Leibo, Sichuan | 19640424 | 0146763 | CDBI |
|  | Leibo, Sichuan | 19640530 | 0146762 | CDBI |
|  | Dujiangyan, Sichuan | 19870512 | 004823 | WCSBG |
|  | Baoxing, Sichuan | 20070626 | 018102 | WCSBG |
